# Marriage of Invalids

**Published:** 1850-01

**Authors:** 


*Marriage of Invalids — Extracted from a manuscript work by Enos Stevens, Examining Agent for the Massachusetts Commissioners for the Prevention and Restoration from Idiocy.*

Of the parents of the idiotic persons, fourteen couples were married in accordance with a medical prescription for their healths and minds; because they were often nervous, irritable, languid, gluttonous, or masturbating, and fast becoming absent minded and forgetful. They were assured by all medical advisers, far and near, that marriage would certainly break up their tendency to masturbation, and probably restore their healths and minds. They argued that their first children would inherit their diseases, and probably die, and carry off the difficulties, leaving the parents well. But in these fourteen cases, the first children did not all die, although they inherited all the scrofulous weaknesses of both their parents, and never improved in mind or body beyond the lowest grades of infantile strength and intelligence. The brains of all their children were as weak as the bodies of their parents, and they are yet living monuments of unhealthy germinations and gestations. Indeed, these parents were relieved of masturbation, but their veneral labors were mostly love's labor lost, while their frequent sexual operations excited all their other diseases and habits to their worst forms and degrees. All these parents were afterwards often insane or lunatic; and all their children, during this state of their health, were either extremely delicate, blind, deaf, puny, deformed, or else idiotic or still-born. But afterwards, some of these parents were correctly advised, and became healthy; and then, when they had lived properly a few years, they had healthy and intelligent children. From all these considerations, and hereditary influences, we perceive the importance, to those who are forming matrimonial alliances, of choosing a spouse who is healthy, intelligent, temperate and industrious; so that when the children resemble the parents, they may not be puny, irritable, lame, deformed, insane nor idiotic. Moreover, when persons are about to become parents, they should take care to be perfectly well, temperate and industrious, so that their children, who resemble them, may not be sickly, gluttonous, intemperate, masturbating, nor insane in some faculties and idiotic in others; until they are reduced to nearly the normal condition by a long course of systematic training in childhood and youth, at a school for the feeble minded.—*Boston Med. and Surg. Journal.*

